# Molecular Surveillance of Dengue in Semarang, Indonesia Revealed the Circulation of an Old Genotype of Dengue Virus Serotype-1

**DOI:** 10.1371/journal.pntd.0002354

**Published:** 2013-08-08

**Authors:** Sukmal Fahri, Benediktus Yohan, Hidayat Trimarsanto, S. Sayono, Suharyo Hadisaputro, Edi Dharmana, Din Syafruddin, R. Tedjo Sasmono

**Affiliations:** 1 Eijkman Institute for Molecular Biology, Jakarta, Indonesia; 2 Health Polytechnic, Jambi Provincial Health Office, Ministry of Health of the Republic of Indonesia, Kotabaru, Jambi, Indonesia; 3 Graduate School in Medicine and Health, Faculty of Medicine, Universitas Diponegoro, Semarang, Indonesia; 4 The Agency for the Assessment and Application of Technology, Ministry of Research and Technology of the Republic of Indonesia, Jakarta, Indonesia; 5 Faculty of Public Health, Universitas Muhammadiyah Semarang, Semarang, Indonesia; Universite de la Mediterranee, France

## Abstract

Dengue disease is currently a major health problem in Indonesia and affects all provinces in the country, including Semarang Municipality, Central Java province. While dengue is endemic in this region, only limited data on the disease epidemiology is available. To understand the dynamics of dengue in Semarang, we conducted clinical, virological, and demographical surveillance of dengue in Semarang and its surrounding regions in 2012. Dengue cases were detected in both urban and rural areas located in various geographical features, including the coastal and highland areas. During an eight months' study, a total of 120 febrile patients were recruited, of which 66 were serologically confirmed for dengue infection using IgG/IgM ELISA and/or NS1 tests. The cases occurred both in dry and wet seasons. Majority of patients were under 10 years old. Most patients were diagnosed as dengue hemorrhagic fever, followed by dengue shock syndrome and dengue fever. Serotyping was performed in 31 patients, and we observed the co-circulation of all four dengue virus (DENV) serotypes. When the serotypes were correlated with the severity of the disease, no direct correlation was observed. Phylogenetic analysis of DENV based on Envelope gene sequence revealed the circulation of DENV-2 Cosmopolitan genotype and DENV-3 Genotype I. A striking finding was observed for DENV-1, in which we found the co-circulation of Genotype I with an old Genotype II. The Genotype II was represented by a virus strain that has a very slow mutation rate and is very closely related to the DENV strain from Thailand, isolated in 1964 and never reported in other countries in the last three decades. Moreover, this virus was discovered in a cool highland area with an elevation of 1,001 meters above the sea level. The discovery of this old DENV strain may suggest the silent circulation of old virus strains in Indonesia.

## Author Summary

We studied dengue disease in Semarang municipality, Central Java, one of the endemic regions in Indonesia. The disease occurred in wide geographical regions which include urban, rural, coastal, and highland areas. All four dengue virus serotypes were found. The infecting serotypes were not associated with disease severities. We also determined the genotype of the circulating viruses. One of the interesting findings was the presence of an old genotype of DENV-1 which has never been reported in the last three decades, which may suggest the silent circulation of this particular genotype in Semarang. These findings offer the first information of the clinical, virological and demographical aspects of the dengue disease in Semarang, Indonesia.

## Introduction

Dengue is one of the most important arthropod-borne viral diseases with large global burden. The disease is caused by dengue virus (DENV), a member of Flaviviridae family, with four distinct serotypes (DENV-1, -2, -3, and -4) circulating in tropical and subtropical regions in the world. DENV is transmitted to human by *Aedes* mosquitoes as vector [1]. Dengue clinical manifestations vary from asymptomatic or mild flu-like syndrome known as classic Dengue Fever (DF) to more severe form known as Dengue Hemorrhagic Fever (DHF) and the potentially fatal Dengue Shock Syndrome (DSS) [2]. DENV genome consists of ∼10.7 kb single-stranded positive-sense RNA genome encoding 3 structural (C, prM/M, E) and 7 non-structural (NS1, NS2A, NS2B, NS3, NS4A, NS4B, NS5) proteins [3]. Similar to other RNA viruses, DENV possess diverse genetic characteristics as shown by the presence of various genotypes within serotypes [4].

Dengue was first reported in Indonesia in 1968 in Jakarta and Surabaya [5]. Up to now, dengue afflicts all the 33 provinces of the vast Indonesian archipelago [6] and become a public health problem annually while periodic major outbreaks occurred such as those in 1998 [7] and 2004 [8]. Nearly 60% of Indonesian people reside in Java island where most of them living in urban areas of big cities where dengue is a problem. However, it has been reported that the disease has also influenced people living in rural areas which probably due to intense people movement [6]. Semarang municipality is a region located in Central Java that is routinely affected by the disease. The region contributes 1.15% of Central Java province with 373.7 km^2^ of areas, divided into coastal and inland areas with various topographical features. The city was inhabited by more than 1.5 million residents. Semarang is listed as top 5 of population number in Central Java with population density of 4,133 per km^2^. In the year of 2011, Semarang region has reported 1,303 dengue cases with 10 fatalities (*Profil Kesehatan Kota Semarang 2011*).

Despite of annual dengue incidence in the region, no detail data of the epidemiology of the disease is present. To fully understand the dengue disease in Semarang municipality, we performed comprehensive dengue surveillance study in Semarang regions, including the Semarang district and Salatiga City, investigating the clinical, virological, and demographical aspects of the disease. The clinical and demographical data of patients were recorded, and the geographical distribution was studied by monitoring the dengue cases both in urban/coastal and rural/highland areas. DENVs were isolated from patients' sera, their serotypes were determined, and their genetic aspects were studied by using phylogenetic and comparative genomic analyses. Correlation between clinical manifestation and virological aspects was also studied.

## Materials and Methods

### Ethics statement

Ethical clearance for this study was obtained from the Dr. Kariadi hospital and Diponegoro University Medical Research Ethics Committees. Dengue-suspected patients from hospitals and primary health care centers were invited to participate in the study and enrolled after written informed consents were obtained from all participants. For minors/children participants, written informed consents were sought from their parent/legal guardians.

### Study site, patient recruitment, and samples collection

**Figure 1:**
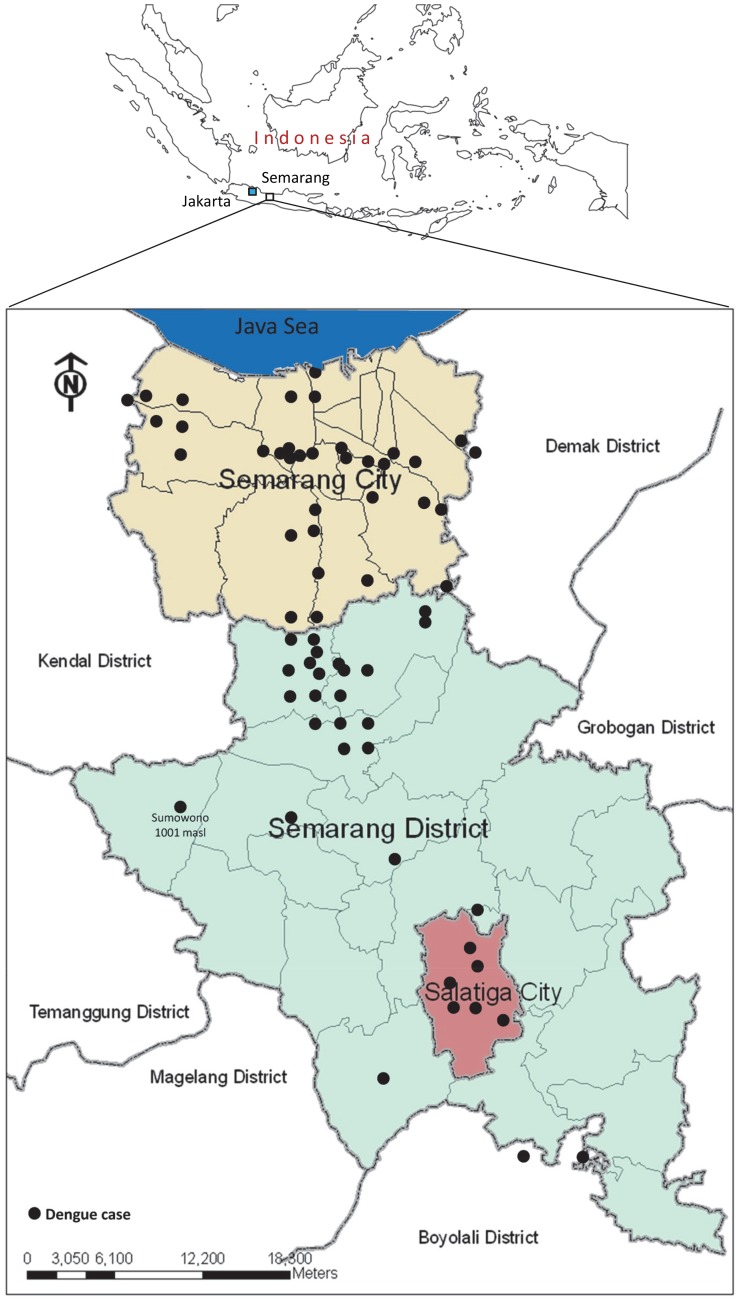
Study area around the Semarang region. Colored areas represent the three districts involved in this study. Black dots represent the locations were the cases occurred.

The study was performed in three regions around Semarang municipality, the capital city of Central Java province, Indonesia during December 2011 until July 2012. The study sites were encompassing both coastal and urban area (Semarang municipality) and rural/highland areas (Semarang district and Salatiga City) with altitude ranged from 0–1,500 meters above sea level (masl). Patients recruitments were conducted at Dr. Kariadi hospital, Dr. Adhyatma hospital, Semarang City hospital, Roemani Muhammadiyah Semarang hospital, Ungaran hospital, Ambarawa hospital, Salatiga City hospital, and primary health care centers (*Puskesmas*), namely Sumowono and Kedungmundu. The geographic coordinates of each patient were recorded using handheld GPS Garmin 72H and mapped (Figure 1).

### Clinical and laboratory examination

Sera from dengue-suspected patients were subjected to serology tests. Serological analysis was performed in sample collection sites using Panbio Dengue Duo Cassette (Alere, Brisbane, Australia) to detect anti-dengue IgG and IgM. Serology test results were confirmed using Panbio Dengue Duo ELISA (Alere) in a laboratory setting, which was also used to determine the primary/secondary infection status of patients. Detection of DENV NS1 antigen was done using Panbio Dengue Early Rapid kit (Alere). Clinical symptoms were documented and laboratory tests including thrombocyte count and hematocrit level were performed as a routine procedure in the hospital. Dengue classification was based on WHO/TDR 2009 guidelines [9].

### RNA extraction and reverse transcriptase-polymerase chain reaction (RT-PCR)

RT-PCR confirmations were performed to detect the presence of DENV in 66 NS1 and/or IgM-positive samples. Virus RNA was extracted from serum samples using QiaAmp Viral RNA Mini kit (Qiagen, Hilden, Germany) according to manufacturer's instructions. DENV nucleic acid detection and serotyping were performed using two steps conventional RT-PCR according to protocol previously described by Lanciotti, *et al.* [10]. Detection and serotyping results were confirmed by quantitative real-time RT-PCR using Simplexa Dengue Molecular Assay performed in 3M Integrated Cycler machine (Focus Diagnostic, Cypress, CA). Detail method of the Simplexa Dengue Molecular Assay was as described by the manufacturer. To determine the positivity of the samples, the cycle threshold (Ct) cut off value of 38 (instead of 40) was used to ensure the strict detection and serotyping of the DENV.

### Virus isolation using cell culture

Serum samples with serological or RT-PCR-positive result were subjected to a maximum of two passages of inoculation in C6/36 (*Aedes albopictus*, mid gut) cell line [11]. Briefly, monolayer of cells was inoculated with 200 µl of sera in 2 ml of 1× RPMI medium supplemented with 2% of Fetal Bovine Serum (FBS), 2 mM of l-glutamine, 100 U/ml of Penicillin, and 100 µg/ml of Streptomycin (all from Gibco-Life Technologies, Carlsbad, CA). Flasks were incubated for 1 hour at 28°C to allow virus attachment. Following the incubation period, inoculation medium was discarded and the medium was replenished with 3 ml of fresh medium. Infected cells were incubated at 28°C for up to 14 days.

### DENV Envelope gene sequencing

DENV genotyping was performed using Envelope (E) gene sequence (1,485 nt in length). DENV RNA was reverse-transcribed into cDNA using Superscript III reverse transcriptase (RT) (Invitrogen-Life Technologies) and DENV-specific primers described elsewhere [12]. The resulting cDNA was then used as template for PCR amplification using *Pfu* Turbo Polymerase (Stratagene-Agilent Technologies, La Jolla, CA). PCR products were purified from 0.8% agarose gel using QIAquick gel extraction kit (Qiagen) and used in cycle sequencing reaction performed using 6 overlapping primers [12] from both strands and BigDye Dideoxy Terminator sequencing kits v3.1 (Applied Biosystems-Life Technologies), following manufacturer's instructions. Purified DNA was subjected to capillary sequencing performed on 3130xl Genetic Analyzer (Applied Biosystems) at the Eijkman Institute sequencing facility. Primers used in genotyping were described elsewhere [12]. Sequence reads were assembled using SeqScape v.2.5 software (Applied Biosystems) with additional manual adjustment performed when manual inspection of the assembly showed some discrepancies. The E protein gene sequences obtained in this study have been deposited in GenBank with accession number KC589008–KC589013 (Supplementary Table S1).

### DENV genotype analysis

For genotype classification, we grouped the isolate sequences with the relevant reference sequences based on classifications by Goncalvez et al. [13], Twiddy et al. [14], and Lanciotti et al. [15] for DENV-1, -2 and -3, respectively. MrBayes was used to construct Bayesian inference phylogenetic trees with mixed model across GTR space model and gamma rates for one million generations with 4 chains, sampled every 1,000 iterations. For evolution studies, we downloaded all publicly available DNA sequences of DENV-1, -2 and -3 from NCBI GenBank as of 12 December 2012. Sample sequences were combined with the downloaded GenBank sequences according to sample's serotypes to create dataset for each dengue serotype. Sequence clustering was performed on each dataset using USEARCH [16]. Multiple alignments resulted from sequence clustering from each cluster containing sample sequences were trimmed to obtain only the alignment representing E protein segment. Sequences without E protein segment were removed from the alignment. We built phylogenetic tree using FastTree [17] for fast approximation with GTR and gamma rate evolution model. We selected 20 closest public sequences from each isolate sequence based on the patristic distance of the FastTree's phylogenetic trees. The multiple alignment of the selected sequences along with the sequence samples were used for phylogenetic reconstruction using Bayesian MCMC method as implemented in BEAST v 1.7.2 [18] using GTR+Γ_4_ model with codon model, relaxed uncorrelated lognormal molecular clock and Bayesian skyline prior, with 60 million generations and sampled for every 1000^th^ iteration.

### Statistical analysis

Statistical analysis was performed using SPSS Statistics software version 11.5 (SPSS Inc., Chicago, IL). Pearson chi-square test was used to correlate the clinical manifestations and DENV serotypes. One-way ANOVA test was used to compare groups of laboratory tests results and DENV serotypes. A probability value of *p*<0.05 was considered statistically significant.

## Results

### Dengue prevalence, demography, and topography

**Figure 2:**
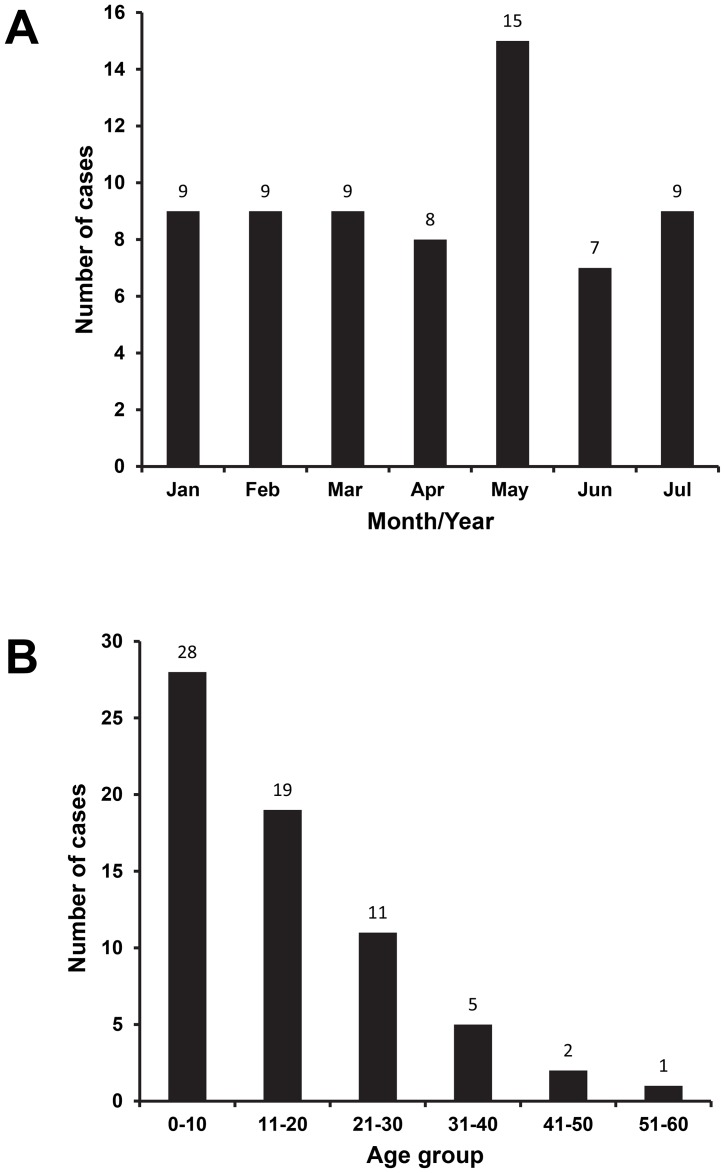
Dengue cases monthly distribution (A) and patients' age (B). All serologically positive dengue patients were grouped according to their hospital admission date and ages.

One hundred and twenty febrile patients were recruited during the course of the study. The dengue cases were equally distributed throughout the month of January–July 2012 with the most cases observed in May (Figure 2A). Of 120 patients, 66 (55%) patients were serologically positive for dengue as determined by IgM/IgG and/or NS1 tests. RT-PCR confirmations were performed to detect the presence of virus on those 66 serum samples and 31 (47%) samples were dengue positive. Of the 66 serologically positive dengue cases, most cases (n = 51 or 77%) were secondary infections, as determined by IgM and IgG ELISA according to kit's manual. The male to female ratio was 1.0 with an average age of 15.98±12.16 (CI 95% 12.99–18.98). Cases occurred predominantly in children aged below 10 year old (42%) (Figure 2B). Among serologically-positive patients, most of them are school children/student (41.7%), followed by working adult (33.3%) and toddler/pre-school children/unemployed adults (25%) (data not shown).

**Figure 3:**
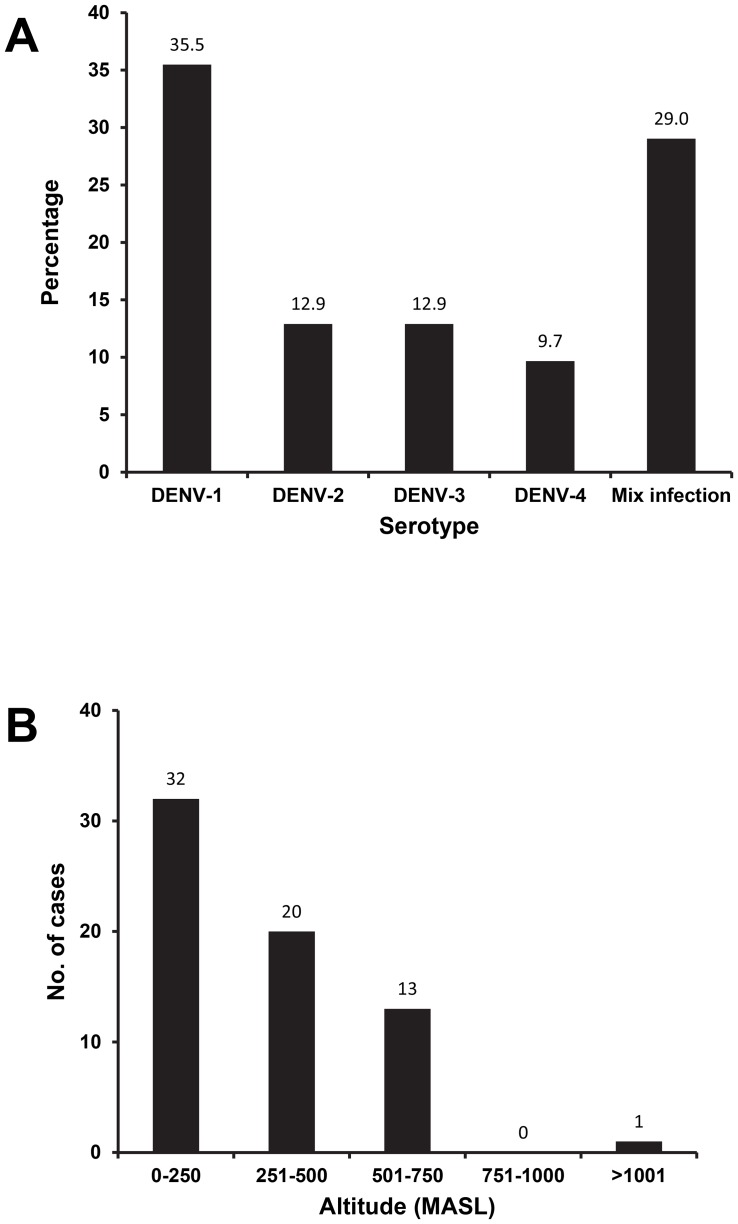
Dengue virus serotype distribution (A) and the altitude of dengue cases (B). Serotypes were determined using both conventional RT-PCR and real-time RT-PCR as described in the Method section. The altitudes of the locations where the cases occurred were recorded using altimeter.

Of the 31 RT-PCR-confirmed dengue cases, serotyping revealed the predominance of DENV-1 (35.5%), followed by DENV-2 (12.9%), DENV-3 (12.9%), and DENV-4 (9.7%). We also observed the presence of multiple infections of different serotypes (29%), of which DENV-1 involved in most of the multiple infections (Figure 3A). These multiple infections were confirmed by Simplexa Dengue Molecular Assay.

Dengue vector distribution is influenced by the local temperature where the mosquitoes circulating. To determine whether topological aspect give account to the dengue cases in Semarang, we performed dengue surveillance encompassing areas with various topological and geographical features. We obtained dengue cases in either coastal or highland areas. Most cases were found in areas with the altitude of 0–250 masl. However, a single case was also found in area with the altitude of 1,001 masl, in which the local temperature in daytime was approximately 24–26°C (Figure 1 and 3B).

### Clinical manifestation

All dengue-confirmed patients underwent clinical examination and laboratory tests at enrollment. The severity of their clinical manifestations was categorized based on WHO 2009 guidelines grading criteria. Of the 66 dengue-confirmed patients, most of them (n = 56 or 84%) were classified as DHF. To assess whether there is correlation between serotype and clinical manifestation, we compiled the patients' data and summarized them in Table 1. Of the 31 cases confirmed by RT-PCR detection, 23 (74%) cases were DHF, and 8 (26%) cases were DSS (Table 1). We did not find any statistically significant correlation between clinical manifestations and medical laboratory examination results of the patients with the infecting DENV serotypes.

**Table 1:** Characteristics of 31 RT-PCR-confirmed dengue patients involved in this study.

Parameters	N	DENV-1	DENV-2	DENV-3	DENV-4	Mix infection	*p* value^c^
**Gender**							0.258
Male	14	5	0	3	1	5	
Female	17	6	4	1	2	4	
**Infection type**							0.919
Primary	6	2	1	1	0	2	
Secondary	25	9	3	3	3	7	
**NS1 antigen detection**							0.231
Positive	25	10	3	3	1	8	
Negative	6	1	1	1	2	1	
**Severity**							0.486
DHF	23	9	3	4	2	5	
DSS	8	2	1	0	1	4	
**Clinical/lab features**							
Fever>37°C	31	11	4	4	3	9	NA
Headache	29	10	4	4	3	8	0.877
Retro-orbital pain	28	9	4	4	3	8	0.716
Myalgia	24	7	3	4	2	8	0.517
Arthralgia	20	6	3	2	2	7	0.784
Nausea	23	7	2	3	3	8	0.417
Loss of appetite	25	9	3	2	3	8	0.461
Rash	13	5	1	2	0	5	0.480
Bleeding	8	2	1	1	0	4	0.556
Leucopenia	14	5	2	2	0	5	0.568
Tourniquet test positive	14	6	0	3	0	5	0.092
Dyspnea	2	1	0	0	0	1	0.877
Abdominal pain	25	8	3	4	3	7	0.900
Mucosal bleeding	10	3	1	1	1	4	0.920
Lethargy	24	8	3	4	3	6	0.594
Restlessness	18	7	0	4	1	6	**0.048**
Drowsiness	16	4	2	3	2	5	0.093
Allergy	2	0	0	2	0	0	**0.006**
Thrombocyte count^a^	NA	77,818±30,619	66,750±22,081	71,000±23,338	69,666±10,016	55,111±28,162	0.484
Hematocrit level^b^	NA	33.3±8.6	35.0±8.4	31.3±9.9	33.7±9.5	32.6±9.4	0.982

a Thrombocyte count in Mean cells/mm^3^ ± SD.

b Hematocrit level in Mean % ± SD.

c Pearson's Chi-squared test, except for thrombocyte count and hematocrit level by One-way ANOVA.

### DENV genotypes and evolution distribution

In order to study the circulating DENV genotypes in Semarang, we performed genotyping analysis based on E gene sequences. The DENV E gene sequences were aligned with reference sequences to generate genotype classifications in each serotype. The resulting phylogenetic trees for the genotype grouping are described in Supplementary Figures.

Of 11 isolates that were serotyped as DENV-1, three viruses were successfully PCR-amplified for their E genes after a single passage in C6/36 cell line. Phylogenetic analysis revealed the grouping of those isolates into two different genotypes, Genotype I (SMG-SE058 and SMG-SE059) and Genotype II (SMG-SE003) based on Goncalvez classification [13] (Supplementary Figure S1). The grouping of Semarang DENV-1 into Genotype II is a new information given this genotype never been found in Indonesia previously. Meanwhile, the Genotype I of DENV-1 is quite commonly found in Indonesia [12], [19].

**Figure 4:**
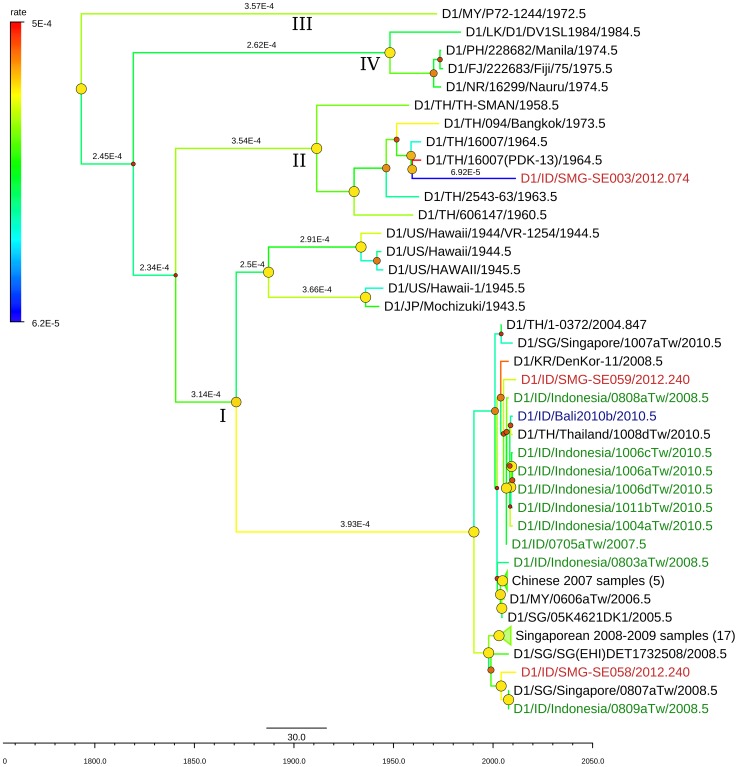
MCC (Maximum Clade Credibility) tree of DENV-1 genotype I and II generated by bayesian inference method as implemented in BEAST using GTR evolution model and gamma parameter rates from the E-protein sequences. The color of branches indicated the rate of evolution of each isolate with blue line for slow rate and red line for faster rate, with values range of 2.72±2.1×10^−4^. The red labels indicated the isolates from Semarang; the green labels indicated other isolates from Indonesia. The dots in the node indicated the posterior probability of that particular cluster, with large yellow dots indicated posterior probability >0.75, medium orange dots for posterior probability between 0.75–0.5, and small red dots for posterior probability <0.5.

We further analyzed the evolutionary history and rate of the DENV-1 from Semarang. Of 20 closely-related sequences of each Semarang isolates from published sequences in GenBank, a total of 56 non-redundant, unique sequences were used for further analysis consisting of mainly isolates from Singapore, China, and imported cases in Taiwan. Because the lack of public sequences for Genotype II, the 20 closely-related sequences for the Genotype II isolate (SMG-SE003) included other genotypes such as Genotype I, III and IV. As shown in Figure 4, this particular isolate was closely related to DENV-1 isolated in Thailand in 1964 [20], which was used as a strain for vaccine development [21]. The mean evolutionary rate of DENV-1 as calculated by BEAST was 2.72×10^−4^ subs/site/year [95% Highest Posterior Density/HPD: 1.24–4.54×10^−4^].

**Figure 5:**
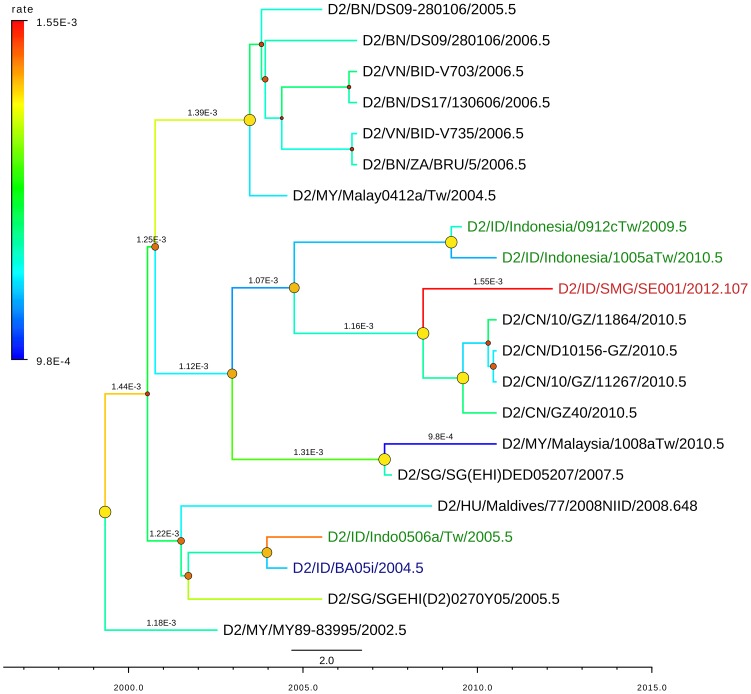
MCC (Maximum Clade Credibility) tree of DENV-2 genotype Cosmopolitan generated by bayesian inference method as implemented in BEAST using GTR evolution model and gamma parameter rates from the E-protein sequences. The color of branches indicated the rate of evolution of each isolate with blue line for slow rate and red line for faster rate, with values range of 11.66±2.1×10^−4^. The red labels indicated the isolates from Semarang; the green labels indicated other isolates from Indonesia. The dots in the node indicated the posterior probability of that particular cluster, with large yellow dots indicated posterior probability >0.75, medium orange dots for posterior probability between 0.75–0.5, and small red dots for posterior probability <0.5.

For DENV-2, we managed to genotype one isolate which was grouped into Cosmopolitan genotype according to Twiddy classification [14] (Supplementary Figure S2). This genotype is commonly found in Indonesia and surrounding countries. The collected 20 closely-related sequences of the Semarang isolate consisted of sequences from Indonesia and neighboring countries such as Singapore, Malaysia and Brunei, as well as other Asian countries such as China, Taiwan, and Vietnam. The Semarang DENV-2 isolate clustered together with Jakarta DENV-2 isolated in 2004 as indicated in Figure 5. The mean rate of evolution of E-protein in this data set was 11.66×10^−4^ subs/site/year [95% HPD: 6.04–17.68×10^−4^].

**Figure 6:**
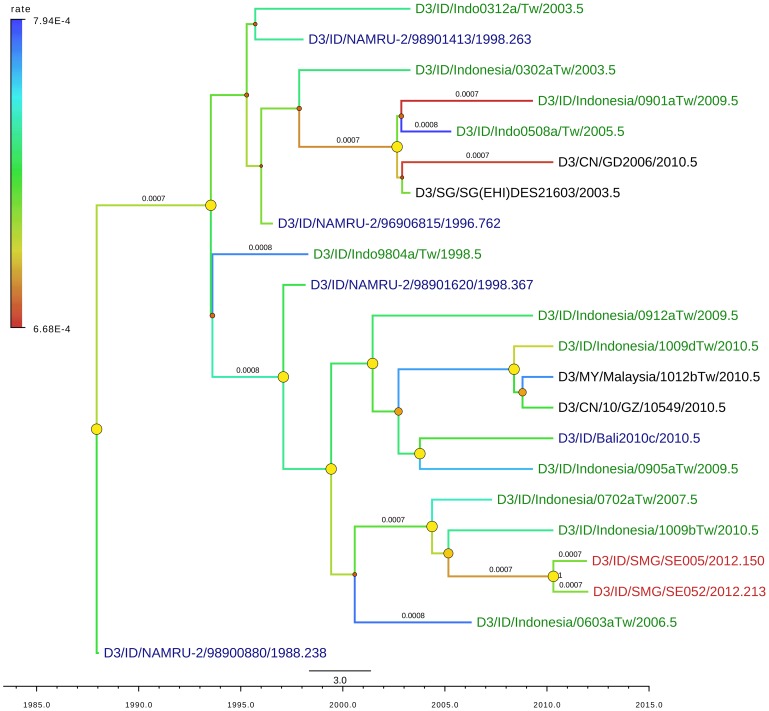
MCC (Maximum Clade Credibility) tree of DENV-3 genotype I generated by bayesian inference method as implemented in BEAST using GTR evolution model and gamma parameter rates from the E-protein sequences. The color of branches indicated the rate of evolution of each isolate with blue line for slow rate and red line for faster rate. The red labels indicated the isolates from Semarang; the green labels indicated other isolates from Indonesia. The dots in the node indicated the posterior probability of that particular cluster, with large yellow dots indicated posterior probability >0.75, medium orange dots for posterior probability between 0.75–0.5, and small red dots for posterior probability <0.5.

We also successfully genotyped two isolates of DENV-3 from Semarang, which were grouped into Genotype I based on Lanciotti classification [15] (Supplementary Figure S3). Figure 6 showed that the majority of 20 non-redundant, unique closely-related sequences from these isolates were Indonesian isolates, either collected previously in other regions in Indonesia such as Yogyakarta (in 1988), Palembang (in 1998) [7], and Jakarta (in 2004) [12] or collected as imported cases such as from Queensland, Australia (of Bali origin in 2010) [22] and from Taiwan. This suggests the endemicity of this genotype in the area. Other isolates from Asian countries such as Singapore, Malaysia and China were also included. The mean evolutionary rate of DENV-3 was 7.24×10^−4^ subs/site/year [95% HPD: 5.08–9.50×10^−4^].

In this study, we also detected three DENV-4 infections (Figure 3A), however, we were not able to PCR-amplify the E gene from the isolates to be used for genotyping.

## Discussion

We described here the clinical, virological, and demographical features of dengue in Semarang, the capital city of Central Java province, and its surrounding regions. The study is somewhat unique as it involved the survey of those features in various regions with different topography, encompassing the coastal, urban/inner city and rural/inland areas, as well as highland areas with the elevation of more than 1,000 m above sea level. Our surveillance was conducted from December 2011 until July 2012. This duration encompasses the rainy and dry season periods, as in Indonesia the wet season is commonly occurred during October–April while the dry season occurred in April–October. The dengue cases were equally detected throughout January to July with the recorded peak in May. Data from Semarang Meteorology Bureau indicated that May to June 2012 was the transition months between wet and dry seasons (data not shown); therefore the high dengue incidence in this month is understandable. The dengue mosquito vectors might still actively breed soon after the decrease of rainfall, since heavy rainfall may wash out the breeding sites while lower rain intensity will maintain the breeding sites.

A total of 120 dengue-suspected patients were recruited in this study. Of this, 66 (55%) patients were serologically confirmed for dengue infection, suggesting that dengue places a considerable burden in the community. Molecular detection revealed the presence of DENV in 31 (47%) patients' sera. Results from serotyping identified the presence of all DENV serotypes in Semarang, with DENV-1 as the predominant serotype, followed by DENV-2, -3, and -4 (Figure 3A). Using a real-time quantitative RT-PCR detection system with a strict standard for detecting the presence of DENV genomes, we observed the presence of multiple DENV infections in nine (29%) out of 31 samples, with DENV-1 involved in most of the multiple infections. This finding further supports the hyper-endemicity of the disease and the predominance of DENV-1. The DENV-1 predominance is currently in place in other cities in Indonesia including in Surabaya [19] and Makassar (Sasmono et al, 2013, submitted for publication). However, historical data of DENV serotype distribution in Indonesia reported the predominance of DENV-2 and -3 in several cities [6], [12]. As there has been a very limited data on dengue serotype distribution in Semarang, we were not able to conclude whether the current serotype replaced the previous serotype predominance.

On the clinical aspect of dengue in Semarang, we documented the clinical symptoms and medical laboratory tests results and grouped the clinical manifestation according to WHO 2009 guideline. In our study, we observed the occurrence of DF, DHF and DSS in dengue-confirmed patients involved in this study. Most of the cases were manifested as DHF, followed by DSS and DF. This finding is understandable as the surveillance was conducted in either health care center or hospital. Based on serological data, most patients (77%) were secondary infection. This indicates sustained disease intensity over a number of years and the endemicity of dengue in the region. There have been studies reporting the association of DENV serotype with clinical manifestation [23]–[25], in which particular serotypes have been correlated with the severity of the disease. To understand the role of each serotype in influencing the clinical outcomes of the disease, we compared the clinical findings of 31 serotyped dengue cases against each serotype. As shown in the Table 1, we did not observe any direct correlation of particular serotype with the disease manifestations. However, we are aware that the small sample number obtained and the unequal distribution of serotypes in this study may not be an ideal basis to draw a conclusion.

In term of geographical feature, this study revealed that the most dengue cases were found in urban area of Semarang City, an area with an elevation of 1–250 masl. The high density population in the area plus the hot and humid weather of the city may give account for the successful transmission of DENV through its *Aedes sp* mosquito vectors. Dengue cases were also reported in Salatiga, a city with a colder weather than Semarang with elevation of 750–850 masl. Probably the most striking finding was the occurrence of one dengue case in Sumowono, a village that is located in the mount Ungaran in the Semarang district. The house where the patient resides has an elevation of 1,001 masl with the average daytime temperature of 24–26°C. To further investigate this rare case (no report of dengue cases in the village in the last 5 years), we conducted vector surveillance and found *Aedes* larvae in used tires and outdoor water containers within the radius of 20–50 m from the house. The infected patient was diagnosed as DHF with thrombocyte count of 51,000/mm^3^ and presented common symptoms of dengue such as fever, headache, nausea, loss of appetite, positive tourniquet test, lethargy, and sleeplessness. The patient was fully recovered.

There is possibility that the dengue infection occurred outside the village but this was negligible, as the patient, a 50 y.o. housewife, was very rarely travelling outside the village because she has been semi-paralyzed due to stroke attack. This finding indicates the virus was transmitted by local *Aedes* mosquito vector and thus suggests the ability of this vector to adapt and circulate in area with higher altitude and colder temperature. Previously, there was a report of dengue outbreak at an area with altitude of 1,700 masl in Guerrero State, Mexico, in 1988 [26]. Therefore the presence of this vector in high altitude area is not impossible. Nevertheless, the occurrence of the dengue cases in this highland area, to the best of our knowledge, represents the first report in Indonesia. A more detail data on the vector surveillance in the study area will be described elsewhere (Fahri et al, 2013, unpublished results).

The genotype of DENV circulating in Semarang was determined by phylogenetic analysis of the E gene of the DENV. For the DENV-1, based on classification by Goncalvez [13], we observed the presence of Genotype I circulating in the region. The SMG-SE058 isolate was clustered with the Singaporean samples isolated in 2008 [27], and had TMRCA (time to most recent common ancestor) around year 1999. The SMG-SE059 isolate was clustered together with Taiwan isolate (0705aTw) in 2007 originated from Indonesia as stated imported case [28], and Korean isolate (DenKor-11) in 2008 from a traveler who visited Indonesia. The TMRCA for this clade is around year 2002. This indicates that strains from these DENV-1 Genotype I clades are likely to have been circulating in Semarang more than a decade. This genotype is currently predominant and common in Indonesia and has been reported to replace the previously predominant Genotype IV [12], [19]. In this study, we did not find the Genotype IV in Semarang area, which may be present but not sampled and genotyped.

The other genotype of DENV-1 that was discovered was the Genotype II, based on Goncalvez classification [13]. This is a novel DENV-1 genotype in Indonesia, as it has never been reported before. This genotype also has never been spotted in other countries in the last three decades. This genotype was isolated from the patient resides in Sumowono village (elevation 1,001 masl) described above. From all publicly available DENV-1 sequences in GenBank, this isolate was found to be very closely related with DENV-1 strain 16007 isolated from patient in Thailand having dengue hemorrhagic fever and shock in 1964 [20], as well as a virus strain which has been undergone serial passages from the parental 16007 isolate (strain 16007(PDK-13)) produced during vaccine development [21]. The evolution rate of Indonesian SMG-SE003 was very slow (6.92×10^−5^ mutation/site/year) compared to the general rate of DENV-1 in this analysis (2.72×10^−4^ mutation/site/year), as indicated by the blue branch line in Figure 4. The high mutation rate of strain 16007(PDK-13), indicated by bright red branch line, was most likely the effect of serial passages process. Another interesting finding related to this strain was that the dengue NS1 antigen test performed in patient's serum was negative, which may raise question if this virus escaped from detection by NS1 diagnostic test. However, the IgM/IgG ELISA confirmed the dengue infection for this sample. Further study is needed to fully understand this finding.

On the origin of this Genotype II strain in Indonesia, one may suspect a possibility of DENV contamination from lab strains during the process of culture, PCR and sequencing. However, this is very unlikely since our lab has never handled and manipulated DENV-1 strains from outside Indonesia (except DENV-1 reference strains Nauru-Western Pacific and Hawaii-USA which do not belong to this genotype). Therefore, this finding reflects the reappearance of an old and unique strain of DENV-1 in Central Java, Indonesia. Considering that SMG-SE003 isolate is closely related to a strain from Thailand isolated in 1964 and has very slow mutation rate, there is a possibility that either this strain has been actually present for a long time and is maintained in nature in a low circulation and infection rate but only sampled now, or that this strain has been dormant and recently emerged into circulation. We are not sure whether this strain will behave like the Thailand strain which caused DHF if it actively re-infecting humans. We are also not sure if it will be spreading and causing epidemic in the region, but given the presence of *Aedes* vector breeding sites in the proximity of isolate origin, the spreading of this strain is possible. Further surveillance is needed to monitor the activity of this strain.

The genotype of DENV-2 circulating in Semarang region was the Cosmopolitan genotype. This genotype is quite common in the region and is widely circulated in India, South East Asia, Africa, the Middle East, and Australia [14]. In Indonesia, this genotype has been circulating since a long time ago and causing outbreaks in 1998 and 2004 [12]. The phylogenetic analysis of the SMG-SE001 indicated that the isolate share common ancestor with some isolates circulating in Taiwan, as imported cases from Indonesia [28], [29], and Guangzhou, China. Semarang is one of the regions in Indonesia that have been supplying workers for countries such as Taiwan; hence dengue cases found in those particular countries might be brought by those workers. Semarang is also one of the regions with high population of Chinese-descendant, with frequent direct flights between Semarang and Guangzhou, China.

The genotypes of DENV-3 isolates from Semarang were clustered in genotype I, which is also common in South East Asia regions. The DENV-3 isolates from Semarang had common ancestor with the isolates from imported cases in Taiwan, which is similar situation as DENV-2 isolate. In particular, the cluster containing these strains had been circulating in Indonesia for more than a decade.

The mean evolution rates of the DENV in this study were within the range observed by other studies, which were in the range of 4.6–11.6×10^−4^ subs/site/year [30]. With the exception of DENV-1 Genotype II isolate SMG-SE003, all rates of individual isolates were within the boundary of the 95% HPD of each corresponding serotypes. This is another indication that this Genotype II isolate warrants a further investigation.

In this study, we were unable to obtain any DENV-4 sequences from Semarang cases. This might be attributed to low viral titers which can only be detected by real-time RT-PCR method, but not sufficient enough for conventional PCR amplification for sequencing purposes. We are aware that there are methods that utilize shorter fragment of E gene that could be used in determining the genotype of our DENV-4, and we might apply this in the future to better understand the genetic aspects of DENV-4 in Semarang.

In conclusion, we have described the clinical, virological, and demographical features of dengue in Semarang in which all serotypes are circulating and highlighted the presence of an old genotype of DENV-1. We also observed the occurrence of dengue in area with high altitude. Altogether, the study suggests the importance of continuous virus surveillance in dengue endemic regions such as Indonesia to better understand the dynamic of the disease.

## Supporting Information

Figure S1 Summary tree of DENV-1 genotype grouping generated by bayesian inference method as implemented in MrBayes from the E-protein sequences. The Semarang isolates (red font) were grouped into genotype I (SMG-SE058 and SMG-SE059) and genotype II (SMG-SE003) based on classification by Goncalves [13]. The posterior probabilities of the clades, indicated as numbers in the node labels, were shown only for major clades.
(PDF)

Figure S2 Summary tree of DENV-2 genotype grouping generated by bayesian inference method as implemented in MrBayes from the E-protein sequences. The Semarang isolate (SMG-SE001) was grouped into Cosmopolitan genotype, based on classification by Twiddy [14]. The posterior probabilities of the clades, indicated as numbers in the node labels, were shown only for major clades.
(PDF)

Figure S3 Summary tree of DENV-3 genotype grouping generated by bayesian inference method as implemented in MrBayes from E-protein sequences. The Semarang isolates (SMG-SE005 and SMG-SE052) were grouped into genotype I, based on classification by Lanciotti [15]. The posterior probabilities of the clades, indicated as numbers in the node labels, were shown only for major clades.
(PDF)

Table S1 Sequenced samples information with the corresponding GenBank accession numbers.
(DOCX)
