# Supplementary figures and images for: Molecular Surveillance of Dengue in Semarang, Indonesia Revealed the Circulation of an Old Genotype of Dengue Virus Serotype-1
Source: PLoS Negl Trop Dis. 2013 Aug 8;7(8):e2354. doi: 10.1371/journal.pntd.0002354 (PMC3738473; doi:10.1371/journal.pntd.0002354)

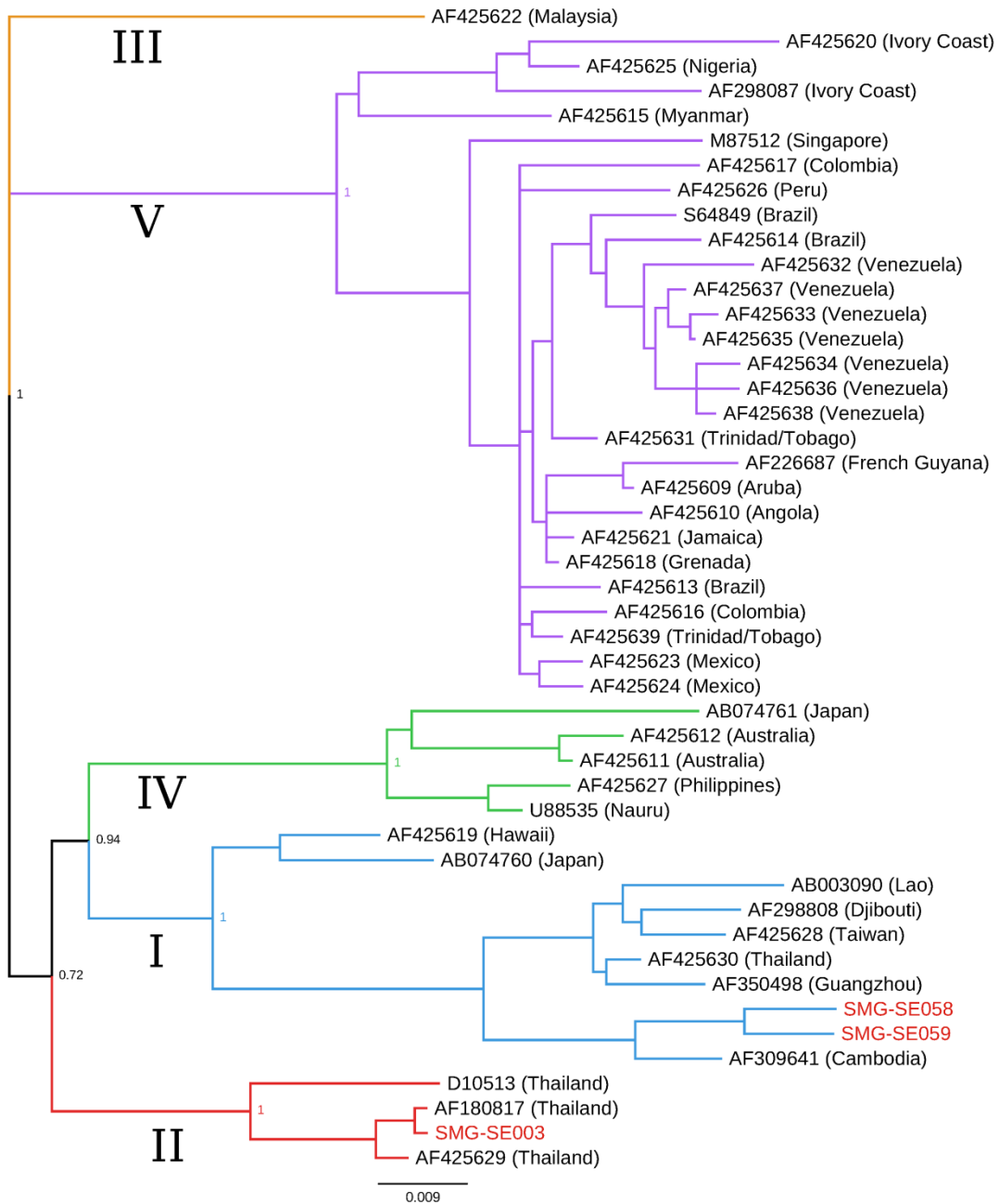

Supplement: Figure S1 — Summary tree of DENV-1 genotype grouping generated by bayesian inference method as implemented in MrBayes from the E-protein sequences. The Semarang isolates (red font) were grouped into genotype I (SMG-SE058 and SMG-SE059) and genotype II (SMG-SE003) based on classification by Goncalves [13]. The posterior probabilities of the clades, indicated as numbers in the node labels, were shown only for major clades. (PDF) [file pntd.0002354.s001.pdf]

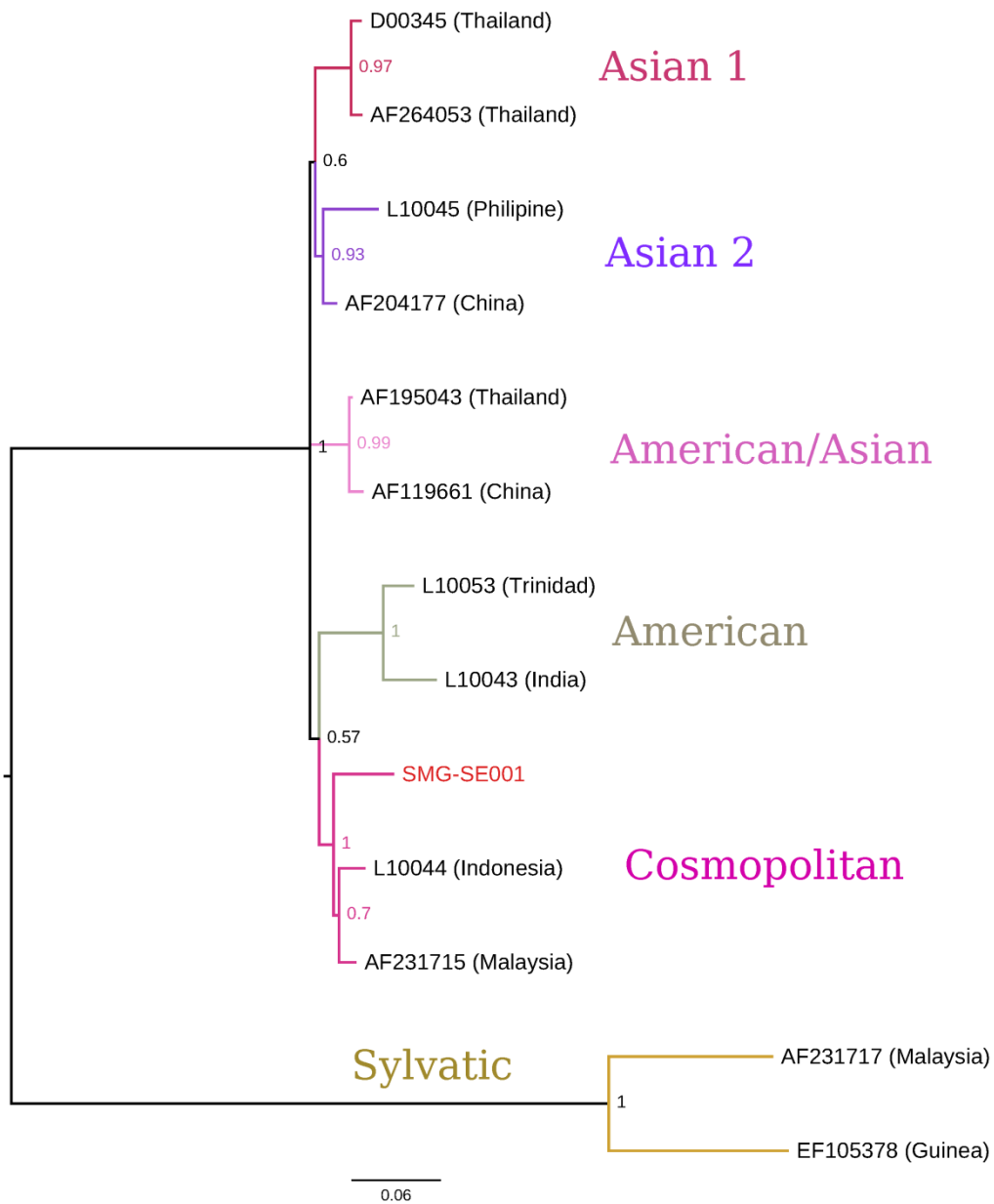

Supplement: Figure S2 — Summary tree of DENV-2 genotype grouping generated by bayesian inference method as implemented in MrBayes from the E-protein sequences. The Semarang isolate (SMG-SE001) was grouped into Cosmopolitan genotype, based on classification by Twiddy [14]. The posterior probabilities of the clades, indicated as numbers in the node labels, were shown only for major clades. (PDF) [file pntd.0002354.s002.pdf]

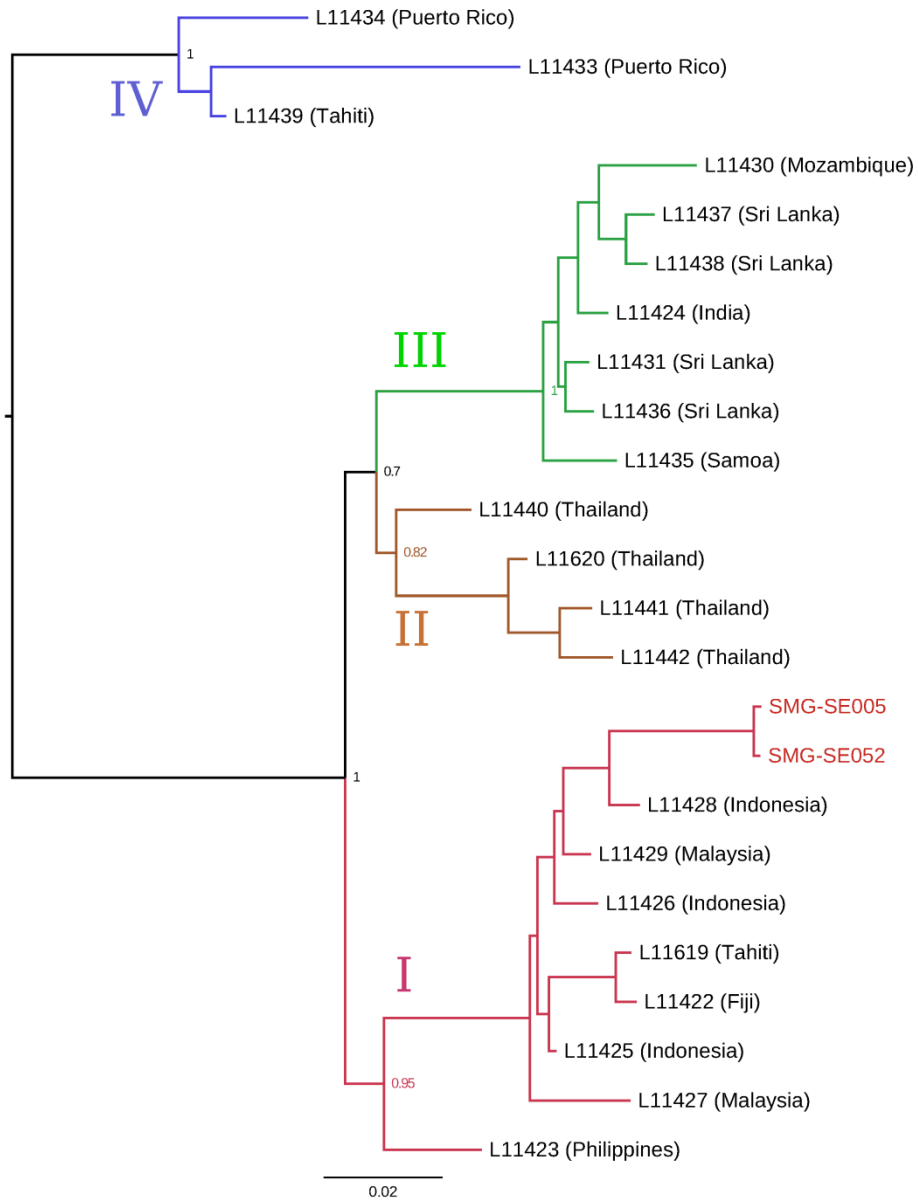

Supplement: Figure S3 — Summary tree of DENV-3 genotype grouping generated by bayesian inference method as implemented in MrBayes from E-protein sequences. The Semarang isolates (SMG-SE005 and SMG-SE052) were grouped into genotype I, based on classification by Lanciotti [15]. The posterior probabilities of the clades, indicated as numbers in the node labels, were shown only for major clades. (PDF) [file pntd.0002354.s003.pdf]
