# Supplementary material for: Molecular Surveillance of Dengue in Semarang, Indonesia Revealed the Circulation of an Old Genotype of Dengue Virus Serotype-1
Source: PLoS Negl Trop Dis. 2013 Aug 8;7(8):e2354. doi: 10.1371/journal.pntd.0002354 (PMC3738473; doi:10.1371/journal.pntd.0002354)
Supplement: Table S1 — Sequenced samples information with the corresponding GenBank accession numbers. (DOCX) [file pntd.0002354.s004.docx]

Supplementary Table S1. Sequenced samples information with the corresponding GenBank accession numbers.

| **Isolate name** | **Serotype** | **Date of isolation** | **Accession number** |
| --- | --- | --- | --- |
| SMG-SE001 | DENV-2 | 09 Feb 2012 | KC589011 |
| SMG-SE003 | DENV-1 | 28 Jan 2012 | KC589010 |
| SMG-SE005 | DENV-3 | 25 Feb 2012 | KC589012 |
| SMG-SE052 | DENV-3 | 19 Mar 2012 | KC589013 |
| SMG-SE058 | DENV-1 | 29 Mar 2012 | KC589009 |
| SMG-SE059 | DENV-1 | 29 Mar 2012 | KC589008 |
